# Inhibition of miR-182-5p Targets FGF9 to Alleviate Osteoarthritis

**DOI:** 10.1155/2023/5911546

**Published:** 2023-03-29

**Authors:** Yang Sun, Sanmao Su, Mengjun Li, Ang Deng

**Affiliations:** ^1^Department of Spine Surgery and Orthopaedics, Xiangya Hospital Central South University, Changsha, China; ^2^Phase I Clinical Trial Center, Xiangya Hospital Central South University, Changsha, China

## Abstract

**Background:**

The pathogenesis of osteoarthritis (OA) is complex and there is no specific drug for treatment. The aim of this study was to identify the molecular targets of OA therapy, focusing on the expression and biological functions of miR-182-5p and its target genes in OA.

**Methods:**

miR-182-5p and fibroblast growth factor 9 (FGF9) were overexpressed or knocked down in IL-1*β*-induced chondrocytes. An OA knee model was performed by surgically destroying the medial meniscus. The gene expression of miR-182-5p and FGF9 was calculated. The protein FGF9 was tested by western blotting. Cell counting kit-8 (CCK8), plate cloning assay, and flow cytometry were conducted to evaluate cell proliferation and apoptosis. The expression of inflammatory factors, tumor necrosis factor-alpha (TNF-*α*), interleukin (IL)-6, and interleukin (IL)-8, was evaluated using enzyme-linked immunosorbent assay (ELISA). Dual-luciferase reporter assays validated the targeting relationship between miR-182-5p and FGF9. Hematoxylin–eosin (HE) and safranin O-fast Green (S–O) staining were utilized to access cartilage damage. Ki67 expression in cartilage was detected using immunohistochemistry (IHC). TdT-mediated dUTP nick-end labeling (TUNEL) assays were used to calculate the apoptosis rate of cartilage.

**Results:**

The expression of miR-182-5p was upregulated, and FGF9 was downregulated in the IL-1*β*-induced chondrocytes. OA chondrocytes proliferation ability in the miR-182-5p mimics group was decreased, and the apoptosis rate and inflammatory factor were increased. Transfection with miR-182-5p inhibitor increased the proliferative ability and decreased the apoptosis rate in the IL-1*β*-induced chondrocytes. Transfection with miR-182-5p inhibitor reversed IL-1*β*-induced inflammatory factor release in chondrocytes. Targeted binding sites existed between miR-182-5p and FGF9. After overexpression of FGF9, the miR-182-5p effect on OA chondrocytes was reversed. The hyaline cartilage thickness and proteoglycan content decreased in OA rats, and this was reversed by miR-182-5p inhibitor treatment.

**Conclusions:**

miR-182-5p expression levels were increased in OA chondrocytes and regulated chondrocyte proliferation, apoptosis, and inflammation by targeting FGF9. miR-182-5p is a potential gene for OA treatment.

## 1. Introduction

Osteoarthritis (OA) is a chronic joint disease. The symptoms often include joint pain, muscle atrophy, and joint deformity [1]. In high-income countries, 80% of people over 65 years of age suffer from OA [2]. OA-related joint pain can lead to limited function, poor quality of sleep, fatigue, and lethargy [3]. Current medications relieve pain and improve function to a certain extent; however, several drugs have been shown to have significant potential side effects [4]. OA pathology includes chondrocyte apoptosis and extracellular matrix (ECM) degradation [5]. The ECM in the articular cartilage is maintained only by chondrocytes, the host cells [6]. Thus, chondrocytes are the primary targets for regulating cartilage degeneration and OA development.

microRNAs (miRNAs/miRs) are short non-coding RNA fragments. Certain miRNAs bind to the 3′-UTRs of target mRNAs to inhibit gene expression [7]. For example, miR-1266-3p can inhibit the development of colon cancer by targeting the 3′-UTR of P4HA3 and inhibiting its expression [8]. Studies showed differential miRNA expression is involved in the ongoing pathophysiology of OA [9, 10]. Using bioinformatics analysis, Huang et al. showed that miR-182-5p expression in OA was upregulated [11]. In addition, miR-182-5p was shown to regulate cell proliferation and the inflammatory response. For example, in triple-negative breast cancer, miR-182-5p regulates cancer cell proliferation and invasion by targeting FBXW7 [12]. Upregulation of miR-182-5p inhibited tumor necrosis factor-alpha (TNF-*α*) induced proliferation and migration of airway smooth muscle cells [13]. miR-182-5p plays a neuroprotective role by inhibiting inflammatory processes in cerebral ischemia–reperfusion injury [14]. miR-182-5p inhibits chondrogenesis through the downregulation of PTHLH [15]. At present, the overall contribution of miR-182-5p in OA is still uncertain and needs further study.

Fibroblast growth factor 9 (FGF9) is a growth factor [16] that regulates osteogenesis and osteoclast formation in bones [17]. Intra-articular injection of exogenous FGF9 has been reported to delay articular cartilage degeneration and aggravate osteophyte formation after traumatic OA [18]. Bioinformatics analysis indicated that FGF9 might be a biomarker of OA with high diagnostic efficiency [19]. In the genetic study of bunions, Weilin Zhang showed that miR-182-5p could bind to FGF9 to regulate bone formation and inhibit osteoblast proliferation [20]. Thus, a miR-182-5p/FGF9 axis may be a vital pathway to block OA progression and needs further exploration.

In this study, we demonstrated a novel mechanism for miR-182-5p regulation in OA. We found that miR-182-5p is elevated in OA chondrocytes, inhibits chondrocyte proliferation, and promotes apoptosis and cartilage degradation by targeting FGF9, as verified by cellular and animal experiments. We demonstrate for the first time the pro-apoptotic function of miR-182-5p in OA, and our study will provide new clues for the diagnosis and intervention of OA.

## 2. Materials and Methods

### 2.1. Cell Culture

Human chondrocytes (CP-H107, Procell Life Science & Technology Co., Ltd.) were grown in Dulbecco's Modified Eagle Media (DMEM) (Gibco; Thermo Fisher Scientific, Inc.). Adherent chondrocytes with 70–80% confluent were cultured in serum-free medium for 12 hours at 37°C. The cells were randomly divided into two groups: a control and an IL-1*β* group. The cells in the IL-1*β* group were stimulated with 10 ng/ml IL-1*β* for 72 hours to simulate OA [21].

### 2.2. Reverse Transcription-Quantitative PCR

Total RNA from human chondrocytes and rat chondrocytes was extracted using TRIzol^®^. The RNA was reverse transcribed to cDNA by reverse transcription using an mRNA reverse transcription kit (CW2569, Cwbio) and miRNA reverse transcription kit (CW2141, Cwbio). Primers were designed and synthesized by Beijing Tsingke Biotechnology, and the sequences are listed in Table 1. Real-time Quantitative PCR (RT-qPCR) was performed using fluorescence quantitative PCR kit (PIKOREAL96, Thermo). The fluorescence intensity during the reaction was recorded in real time. The gene expression was calculated by 2^−*ΔΔ*Ct^ method compared with *β*-actin, U6, or 5S.

### 2.3. Western Blotting

Radioimmunoprecipitation (RIPA) solutions were utilized to extract total proteins. Sodium dodecyl sulfate-polyacrylamide gel electrophoresis (SDS-PAGE) was performed to separate proteins, and the resolved proteins were transferred to a membrane. After transfer, the membrane was immersed in 5% defatted milk for 90 minutes. The membrane was incubated with primary antibodies FGF9 (ab206408, Abcam) and *β*-actin (66009-1-Ig, Proteintech) at 4°C overnight. The membrane was then incubated with the secondary antibodies HRP goat anti-mouse IgG (1 : 5000, SA00001-1, Proteintech) or HRP goat anti-rabbit IgG (1 : 6000, SA00001-2, Proteintech) for 90 minutes. SuperECL Plus luminescence solution (AWB0005, ABiowell) was used to visualize the signals. Protein expression was normalized with *β*-actin expression.

### 2.4. Plasmid Construction and Cell Transfection

To study the role of miR-182-5p in OA development, CP-H107 cells were separated into five groups: control, IL-1*β*, NC, miR-182-5p mimics, and miR-182-5p inhibitor group. In order to study the mechanism of miR-182-5p, CP-H107 cells were divided into four groups: mimics NC, miR-182-5p mimics, miR-182-5p mimics + oe-NC, and miR-182-5p mimics + oe-FGF9 group. All mimics, inhibitor, and its NC were designed and constructed by Geneseed (Guangzhou, China). Lipofectamine 2000 (Invitrogen) was utilized as a co-transfection agent to transfect oligostranded RNA into the corresponding groups of chondrocytes. After 48 hours of transfection, the cells were treated with 10 ng/mL IL-1*β* for 72 hours (except for the control group), and the cells were collected for further experiments.

### 2.5. Cell Counting Kit-8

Cell counting kit-8 (CCK8) assays (NU679, DOJINDO) were sued to detect cell viability. CP-H107 cells were digested into single cells using a trypsin digestion solution. The cells were inoculated into 96-well plates at a concentration of 1 × 10^4^/100 *μ*l in triplicate. Each group of cells was inoculated into three wells. After 0 hour, 48 hours, or 72 hours, the medium was replaced with CCK8 working solution (medium containing CCK8 solution). The cells were then incubated in the 5% CO_2_ incubator at 37°C for 4 hours.

### 2.6. Plate Cloning Assay

Cell suspensions in serum-free basal medium were prepared, seeded in 6-well plates (1 × 10^3^ cells/well), and shaken well. Then the cells were cultured in the 37°C, 5% CO_2_ incubator. After 3 weeks of culture, the culture medium was discarded. Cells were fixed with 4% paraformaldehyde (P0099, Beyotime) for 30 minutes and then stained with 0.5% crystal violet (C0121, Beyotime) for 5 minutes. The cells were photographed and counted.

### 2.7. Flow Cytometry

Precooled anhydrous ethanol was utilized to immobilize the cells. On the second day, ethanol was removed by centrifugation at 1500 rpm for 5 minutes. Then cells were dyed with 150 *μ*l working solution (50 mg/ml propidium iodide, 0.1 mg/ml RNase, and 0.2% Triton X-100) at 4°C for 30 minutes under dark conditions [22]. The cell cycle was analyzed using a flow cytometer (A00-1-1102, Beckman). A total of 1 × 10^4^ cells were analyzed in each forward scatter (FSC)/side scatter (SSC) plot, and adhesive cells and debris were removed on a case-by-case basis by setting the gates as appropriate. An Annexin V-FITC Apoptosis Detection Kit (KGA108, KeyGen) was used to calculate the apoptosis rate of cells according to the manufacturer's protocol. After 10 minutes of reaction, the cells were analyzed using flow cytometry.

### 2.8. Targetscan and Dual-Luciferase Reporter Assays

Targetscan (https://www.targetscan.org/vert_71/) was used to predict the binding between miR-182-5p and the 3′UTR of FGF9. The human FGF9 gene 3′UTR double luciferase reporter plasmid pHG-MirTarget-FGF9-3U (wild type, WT) and the human FGF9 gene 3′UTR double luciferase reporter plasmid pHG-MirTarget-FGF9-3U-Mut (mutant, Mut) were purchased from the Changsha HonorGene. The plasmid was extracted and its concentration was 1 *μ*g/*μ*l. A dual-luciferase Detection Kit (E1910) was purchased from Promega Corporation and used according to the manufacturer's protocol. The luciferase fluorescence signal was measured using a chemiluminescence detector (GloMax 20/20, Promega).

### 2.9. In Vivo Studies

A total of 20 male SD rats (200–250 g) were purchased from Hunan Slyke Jingda Animal Research Center and separated into four groups: Sham, OA, inhibitor NC, and miR-182-5p inhibitor groups. In OA, inhibitor NC, and miR-182-5p inhibitor groups, an OA knee model was performed by surgical destruction of the medial meniscus (DMM) [23]. In the Sham group, the medial meniscus tibial ligaments were left intact. One week after the operation, the rats in the inhibitor NC group were treated with 10 *μ*l inhibitor NC by intra-articular injection twice a week for 4 weeks. The miR-182-5p group was injected with miR-182-5p inhibitor through intra-articular injection twice a week for 4 weeks. Rats in the Sham and OA group were injected with an equal volume of medium [23]. At the end of the experiments, the rats were euthanized with an overdose of 1% sodium pentobarbital (150 mg/kg). The death of the rats was confirmed by a lack of a heart rate and respiratory arrest. All animal experiments were approved by the Animal Ethical and Welfare Committee of Xiangya Hospital Central South University and performed in accordance with the guidelines of the Animal Care and Ethics Committee.

### 2.10. Enzyme-Linked Immunosorbent Assay

TNF-*α* (cat. no. KE00154, ProteinTech Group, Inc.), interleukin (IL)-6 (cat. no. KE00139, ProteinTech Group, Inc.), and interleukin (IL)-8 (cat. no. CSB-E04641h, CUSABIO) ELISA (enzyme-linked immunosorbent assay) kits were used to determine the levels of TNF-*α*, IL-6, and IL-8 in the supernatant according to manufacturer's instructions. TNF-*α* (cat. no. CSB-E11987r, CUSABIO), IL-6 (cat. no. CSB-E04640r, CUSABIO), and IL-8 (cat. no. ml002885, Mlbio) ELISA kits were used to determine the cartilage levels of TNF-*α*, IL-6, and IL-8 according to manufacturer's instructions.

### 2.11. Hematoxylin–Eosin (HE) Staining

The rat cartilage tissue was cut into 2 *μ*m thick sections. Sections were baked at 60°C for 12 hours and then dewaxed and rehydrated using xylene and ethanol, respectively. Sections were stained with hematoxylin and eosin for 10 minutes and 5 minutes, respectively, and subsequently, placed in xylene for 10 minutes, twice. Finally, sections were sealed and observed using a microscope (BA410T, Motic).

### 2.12. Safranin O–Fast Green (S–O) Staining

The rat cartilage tissue was cut into 2 *μ*m thick sections. Sections were incubated with solid green for 5 minutes and washed with 1% acetic acid. Then, sections were stained with saffron for 30 seconds. Subsequently, sections were soaked in xylene for 20 minutes, and sealed and observed using a microscope.

### 2.13. Immunohistochemistry

The rat cartilage tissue was cut into 2 *μ*m thick sections. The sections were heated for 20 minutes to repair the antigens, and subsequently incubated with 1% periodate acid for 10 minutes to inactivate endogenous enzymes. Next, the tissues were incubated with primary antibody Ki67 (ab16667, 1 : 200, Abcam) at 4°C overnight, followed by incubation with the appropriate secondary antibody HRP-labeled anti-rabbit IgG polymer (PV-9001, ZSGB-BIO). A DAB working solution was used to develop the signal. Cells were counterstained with hematoxylin for 10 minutes, and ethanol was used to dehydrate the sections. Finally, the sections were sealed and imaged using a microscope.

### 2.14. TdT-Mediated dUTP Nick-End Labeling

The rat cartilage tissue was made into 2 *μ*m thick sections. TUNEL Apoptosis Detection Kit (FITC) (40306ES20, YEASEN) was used to detect apoptosis in tissue after section hydration. All operations strictly followed the manufacturer's instructions. Subsequently, 4′,6-diamidino-2-phenylindole (DAPI) working solution was used to counterstain the tissues. Sections were blocked with glycerin buffer and imaged using a microscope.

### 2.15. Statistical Analysis

All data of this paper were statistically calculated by GraphPad Prism 8. A student *t*-test was utilized to compare differences between the two groups. Differences between multiple groups were analyzed by one-way Analysis of Variance (ANOVA) accompanied by Tukey multiple comparisons posttest. *P* < 0.05 was considered statistically significant.

## 3. Results

### 3.1. The Expression of miR-182-5p and FGF9 in OA Chondrocytes Was Changed

**Figure 1:**
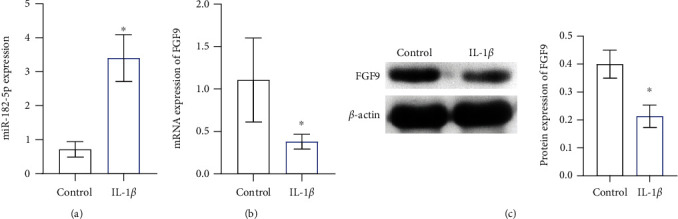
The expression of miR-182-5p and FGF9 in OA chondrocytes was changed. The gene expression of (a) miR-182-5p and (b) FGF9. (c) The protein expression of FGF9. ∗*P* < 0.05 vs. control.

To study the changes in the expression of miR-182-5p and FGF9 in chondrocytes, we stimulated CP-H107 for 72 hours with IL-1*β* to simulate OA chondrocytes.  Figure 1(a) shows that miR-182-5p expression was significantly increased in IL-1*β*-stimulated cells. Additionally, compared with the control group, the FGF9 mRNA and protein expression were decreased in IL-1*β*-stimulated cells (Figures 1(b) and 1(c)). This suggested that miR-182-5p and FGF9 might impact the occurrence and development of OA in a significant way.

### 3.2. miR-182-5p Inhibited Chondrocyte Proliferation and Promoted Apoptosis

**Figure 2:**
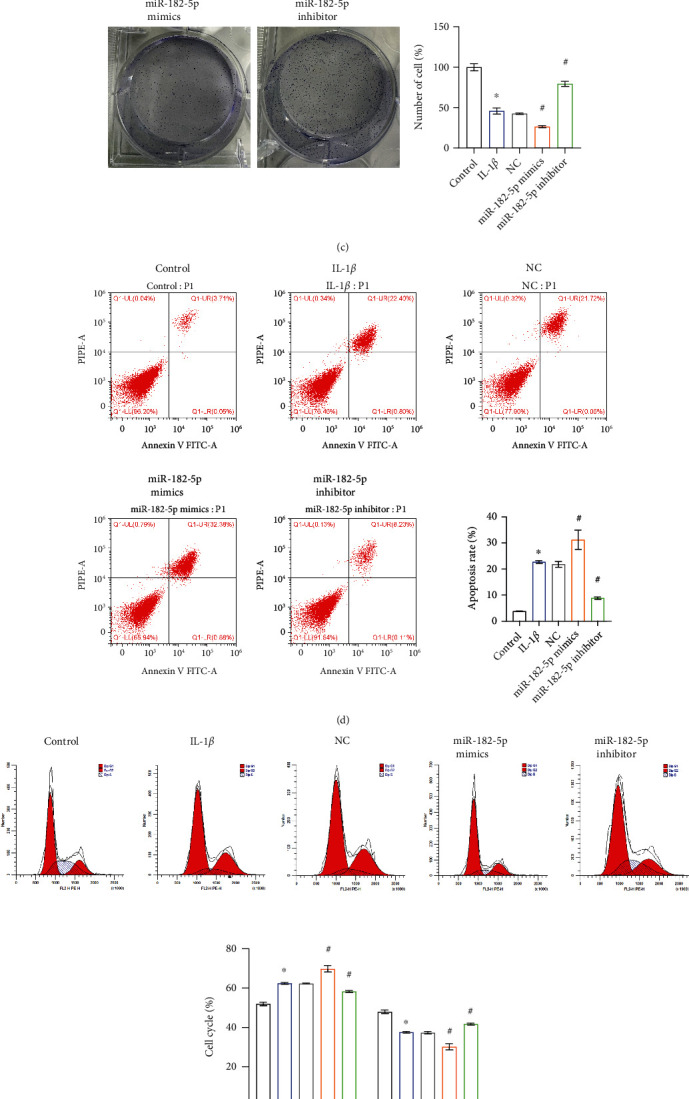
miR-182-5p inhibited chondrocyte proliferation and promoted apoptosis. (a) The gene expression of miR-182-5p. (b) CCK8 was used to determine the cell viability. (c) Photos and analysis of plate cloning of each group of cells. Flow cytometer was utilized to test the (d) apoptosis and (e) cell cycle. (f) The expression of inflammatory factors (TNF-*α*, IL-6, and IL-8) was evaluated by ELISA. ∗*P* < 0.05 vs. control. ^#^*P* < 0.05 vs. NC.

To explore the effect of miR-182-5p on chondrocytes, we knocked down or overexpressed miR-182-5p in chondrocytes. Reverse transcription-quantitative PCR (RT-qPCR) result showed that miR-182-5p was successfully knocked down or overexpressed in the corresponding groups (Figure 2(a)). After 72 hours of culture, compared with the NC group, the cell viability of the miR-182-5p mimics group was decreased, while the cell viability of the miR-182-5p inhibitor group had increased (Figure 2(b)). As shown in Figure 2(c), the cell viability in the miR-182-5p mimics group was significantly lower than that in the NC group. In comparison, cell viability in the miR-182-5p inhibitor group was significantly higher than that in the NC group. Similarly, the apoptosis rate was significantly increased in the miR-182-5p mimics group, whereas the reverse was seen in the miR-182-5p inhibitor group (Figure 2(d)). The G2 + S phase cells were significantly reduced after miR-182-5p mimics treatment, while in the miR-182-5p inhibitor group, it had increased (Figure 2(e)). As shown in Figure 2(f), the levels of the inflammatory factors TNF-*α*, IL-6, and IL-8 in the supernatant of CP-H107 cells were significantly increased after IL-1*β* treatment. Transfection with miR-182-5p mimics exacerbated IL-1*β*-induced inflammatory factor release in CP-H107 cells, whereas transfection with miR-182-5p inhibitor reversed IL-1*β*-induced inflammatory factor release in CP-H107 cells. In general, miR-182-5p reduced chondrocyte viability, inhibit proliferation, promote apoptosis and inflammation, and alter the cell cycle.

### 3.3. miR-182-5p Targeted FGF9 Expression in Chondrocytes

**Figure 3:**
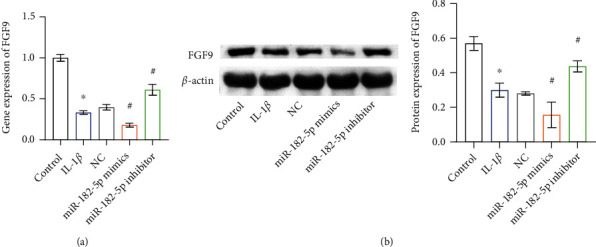
miR-182-5p targeted FGF9 expression in chondrocytes. (a) The gene expression of FGF9. (b) The protein expression of FGF9 was analyzed by WB. ∗*P* < 0.05 vs. control. ^#^*P* < 0.05 vs. NC.

**Figure 4:**
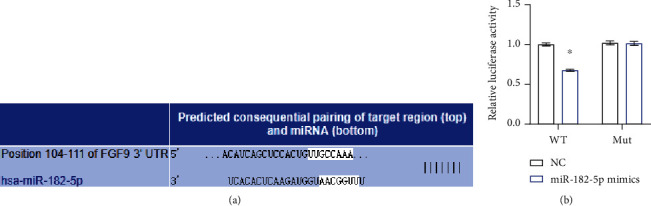
miR-182-5p was targeted to FGF9. (a) Targetscan revealed the binding sites of miR-182-5p and FGF9. (b) The targeting relationship of miR-182-5p and FGF9. ∗*P* < 0.05 vs. NC (WT).

Next, we investigated the influence of miR-182-5p on FGF9 expression. At the mRNA and protein levels, the FGF9 expression in the miR-182-5p mimics group was lower than in the NC group. Meanwhile, compared with the NC group, the FGF9 expression in the miR-182-5p inhibitor group was increased (Figures 3(a) and 3(b)). This indicated that miR-182-5p inhibited FGF9 transcription and translation. Targetscan prediction predicted the presence of a binding site for miR-182-5p in the 3′UTR region of FGF9 (Figure 4(a)). To further explore the interaction between miR-182-5p and FGF9, we conducted a dual-luciferase reporting assay. Compared with NC (FGF9-WT), the relative luciferase activity in the miR-182-5p mimics group (FGF9-WT) was reduced (Figure 4(b)). However, there was no difference in relative luciferase activity among different treatments in FGF9-MUT cells. This showed that miR-182-5p could target the 3′UTR region of FGF9 to inhibit its transcription and translation.

### 3.4. miR-182-5p Targeting FGF9 Inhibited Chondrocyte Proliferation and Promoted Apoptosis

**Figure 5:**
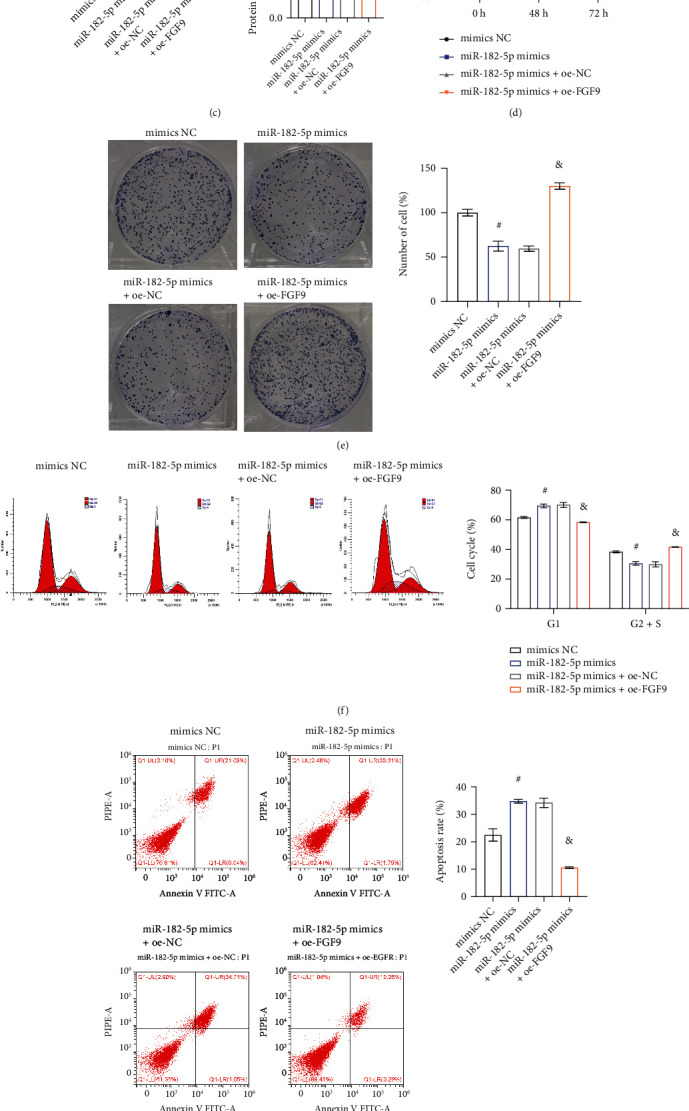
miR-182-5p targeting FGF9 inhibited chondrocyte proliferation and promoted apoptosis. The gene expression of (a) miR-182-5p and (b) FGF9. (c) The protein expression of FGF9. (d) Cell viability was monitored by CCK8. (e) Plate cloning of cells. The (f) cell cycle and (g) apoptosis was captured by flow cytometry. (h) The expression of inflammatory factors (TNF-*α*, IL-6, and IL-8) was evaluated by ELISA. ^#^*P* < 0.05 vs. mimics NC. ^&^*P* < 0.05 vs. miR-182-5p mimics + oe-NC.

To verify whether miR-182-5p inhibited chondrocyte proliferation by regulating FGF9, we conducted a rescue experiment to observe the effect. After transfection of miR-182-5p mimics in chondrocytes, miR-182-5p expression was significantly higher at the mRNA level. However, the mRNA expression of FGF9 was significantly decreased. But, the above effects were rescued when FGF9 was overexpressed in the cells transfected with miR-182-5p (Figures 5(a) and 5(b)). Then, we verified the decreased protein expression of FGF9 in chondrocytes after transfection with miR-182-5p using western blot. Interestingly, when FGF9 was overexpressed in the cells transfected with miR-182-5p, the protein expression of FGF9 was relatively elevated (Figure 5(c)). CCK8 and plate cloning assay reflected overexpression of miR-182-5p reduced cell proliferation. FGF9 overexpression reversed the effect of miR-182-5p (Figures 5(d) and 5(e)). Interestingly, compared with the miR-182-5p mimics + oe-NC group, the miR-182-5p mimics + oe-FGF9 group exhibited a lower proportion of cells in the G1 phase and an increased proportion of cells in the G2 + S phase (Figure 5(f)). Meanwhile, the apoptosis rate of the miR-182-5p mimics + oe-FGF9 group was less than that of the miR-182-5p mimics + oe-NC group (Figure 5(g)). ELISA results showed that overexpression of FGF9 reversed the increase in inflammatory factors levels caused by overexpression of miR-182-5p (Figure 5(h)). These results suggested that miR-182-5p could inhibit chondrocyte proliferation, and promote apoptosis and inflammation. Moreover, FGF9 reversed the effect of miR-182-5p.

### 3.5. Inhibition of miR-182-5p Alleviated the Process of OA in Rats

**Figure 6:**
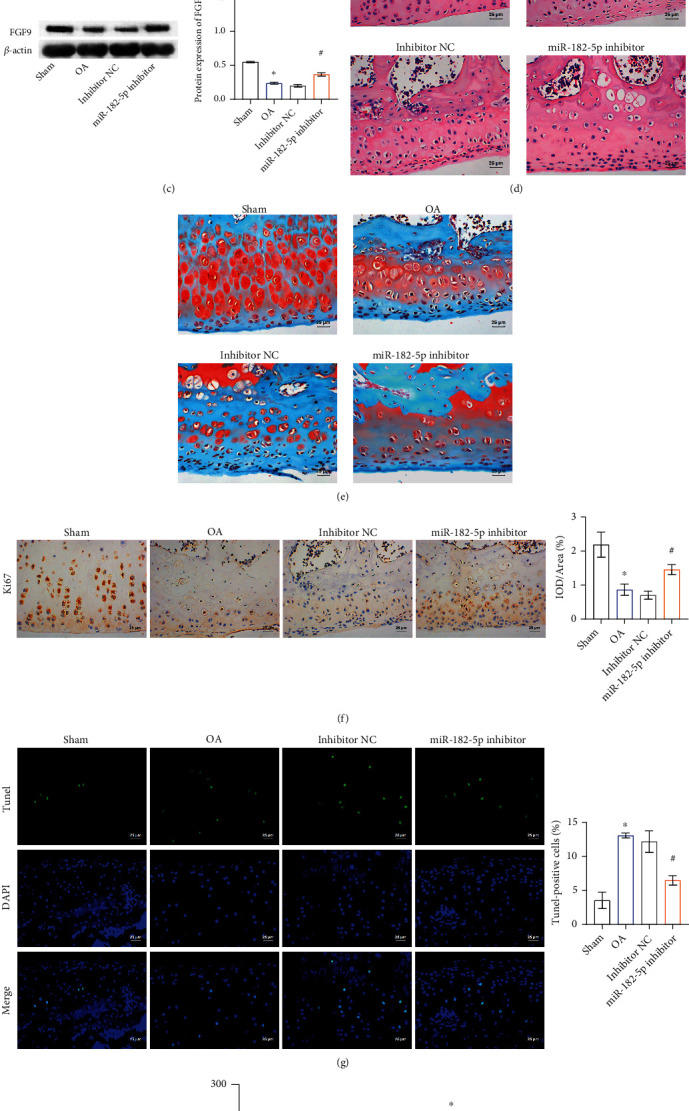
Inhibition of miR-182-5p alleviated the process of OA in rats. RT-qPCR was conducted to validate the gene expression of (a) miR-182-5p and (b) FGF9. (c) The protein expression of FGF9. (d) HE showed the structure of cartilage tissues. (e) Proteoglycans in cartilage tissue were exposed by S–O staining. (f) IHC was adopted to capture the expression of Ki67. (g) The rate of apoptosis was measured by TUNEL. (h) The expression of inflammatory factors (TNF-*α*, IL-6, and IL-8) was evaluated by ELISA. Magnification = 400. Scale bar = 25 *μ*m. ∗*P* < 0.05 vs. Sham. ^#^*P* < 0.05 vs. inhibitor NC.

Finally, we performed *in vivo* animal experiments. The result of RT-qPCR indicated that miR-182-5p expression was successfully inhibited in the miR-182-5p inhibitor group of rats (Figure 6(a)). Additionally, the mRNA and protein expression levels of FGF9 in the miR-182-5p inhibitor group were increased (Figures 6(b) and 6(c)). To determine the progression and severity of OA, the proteoglycan and structural degeneration of articular cartilage were assessed under the microscope following safranin O-fast Green (S–O) and hematoxylin–eosin (HE) staining. HE staining showed that the thickness of hyaline cartilage decreased and articular chondrocytes were disordered in the OA group. miR-182-5p inhibitor treatment significantly reversed OA symptoms (Figure 6(d)). When cartilage was damaged, glycoproteins in the cartilage were released, resulting in uneven distribution of matrix components, and light to no S–O staining was observed. The proteoglycan content of cartilage in the OA group was reduced, and cartilage erosion was observed. The mice in the miR-182-5p inhibitor group showed proteoglycan retention and less cartilage erosion compared with NC-treated rats (Figure 6(e)). Ki67 was widely used as a proliferation indicator in clinical practice [24]. As shown in Figure 6(f), the proliferation rate of chondrocytes in OA rats was lower than that in the Sham group. Compared with the inhibitor NC group, the proliferation rate of chondrocytes in the miR-182-5p inhibitor group was higher. TdT-mediated dUTP nick-end labeling (TUNEL) assays showed that the apoptosis rate of chondrocytes in the OA group was higher than that in the Sham group, and miR-182-5p inhibitor treatment reversed this (Figure 6(g)). ELISA results showed that the levels of inflammatory factors in the cartilage of the OA group were higher than that in the Sham group, and miR-182-5p inhibitor treatment reversed this trend (Figure 6(h)). Together, these results indicated that miR-182-5p inhibitor target FGF9 to promote chondrocyte proliferation, inhibit chondrocyte apoptosis and inflammation, and ultimately hinder the development of OA in rats.

## 4. Discussion

OA is the most common joint disease and the primary cause of functional impairment and disability. The cost of treatment and the socio-economic burdens are significant. However, there are no effective methods to block the progression of OA at present, which requires clinical treatment according to the different reactions of different parts of the disease [25]. miR-182-5p participates in cell function and inflammatory response, which might provide a new approach for OA treatment. Currently, the function, target, and molecular mechanism of miR-182-5p on OA remain incompletely understood. In our paper, miR-182-5p targets FGF9 to inhibit OA chondrocyte proliferation. *In vivo*, inhibition of miR-182-5p alleviated the development of OA in rats. This indicated that miR-182-5p is a promising target for OA.

Previous studies have shown that miRNAs have a large potential in assisting early diagnosis and effective treatment of OA [26]. Our results revealed that miR-182-5p expression was increased in IL-1*β*-stimulated chondrocytes. In advanced OA, chondrocytes are reduced, usually accompanied by lacunar empty. Apoptosis obviously occurs in OA cartilage [6]. Recently, more and more evidence has revealed miR-182-5p regulates apoptosis and inflammatory response [27]. miR-182-5p inhibits ox-low-density lipoprotein (LDL)-induced vascular smooth muscle cell proliferation by targeting pregnancy associated plasma protein-A (PAPPA) [28]. miR-182-5p could inhibit endometrial stromal cell proliferation, migration, invasion, and inflammation through the NF-*κ*B signaling pathway in endometriosis [29]. What's more, miR-182-5p exerts a pro-apoptotic effect in cardiomyocytes under hypoxic conditions through the downregulation of cytokine-induced apoptosis inhibitor 1 (CIAPIN1) [30]. Our study found that transfection with miR-182-5p inhibitor reversed the apoptosis and inflammation in chondrocytes induced by IL-1*β*, and promoted proliferation. Moreover, miR-182-5p mimics significantly inhibited chondrocyte proliferation, promoted apoptosis and inflammation, and may thus play an essential role in OA. To the best of our knowledge, this is the first time such a relationship has been reported.

FGF9 regulates the cycle and apoptosis of various cells. For example, FGF9 promotes p38 mitogen‐activated protein kinase (MAPK) signaling mediated mouse spermatogonial stem cell proliferation in mouse testis [31]. Additionally, FGF9 is a well-known factor regulating bone development, which can inhibit osteogenesis and promote osteoclast formation [17]. The expression of FGF9 was significantly reduced in OA chondrocytes. This may be related to the proliferation and apoptosis of OA chondrocytes. More interestingly, we indicated that miR-182-5p bound to FGF9 mRNA to inhibit its expression in chondrocytes. Previous studies have also demonstrated miR-182 can target FGF9 in a variety of cells. miR-182 inhibits the proliferation and migration of Schwann cells by targeting FGF9 and neurotrimin (NTM), respectively, in the early stages of sciatic nerve injury [32]. miR-182 blocks the dedifferentiation of vascular smooth muscle cells through FGF9/platelet-derived growth factor receptor *β* (PDGFR*β*) signaling [33]. However, the mechanism of action of miR-182-5p and FGF9 in OA has not been investigated. Our study confirmed the targeted inhibition of FGF9 by miR-182-5p in OA chondrocytes. Subsequently, we verified our conjecture using rescue experiments in which FGF9 was overexpressed. FGF9 was found to reverse the effect of miR-182-5p in the IL-1*β*-induced chondrocytes. This suggested that miR-182-5p inhibited chondrocyte proliferation by targeting FGF9. We have demonstrated for the first time the mechanism of miR-182-5p regulation in OA.

Proteoglycan is an important structural component of the chondrocyte ECM and provides expansion pressure for cartilage [34]. One hallmark of OA is the degradation of articular cartilage proteoglycans [35]. Natural proteoglycan levels are significantly reduced in OA, and studies have suggested that biomimetic proteoglycans may have potential as a treatment for OA [36]. In our study, the content of proteoglycan in the cartilage of rats in the OA group was significantly reduced, which was reversed by miR-182-5p inhibitor treatment. In cartilage, chondrocytes are responsible for the biogenesis and maintenance, which consists of proteins, glycoproteins, and proteoglycans [37]. Chondrocyte apoptosis is closely related to the severity of cartilage damage and matrix depletion in OA joint [38]. Reducing chondrocyte death may hold significant promise for future OA treatment. Additionally, our paper reflected that miR-182-5p inhibitor treatment hindered apoptosis in OA articular chondrocytes. This revealed miR-182-5p is a vital gene for OA.

Overall, miR-182-5p inhibitor treatment inhibited chondrocyte apoptosis, reduced inflammation, and assisted in maintaining proteoglycan levels in joint tissue, thereby alleviating the pathophysiology of OA. This effect was achieved by targeting FGF9. This discovery further advances our understanding of the pathological mechanisms underlying the development of OA and may lead to novel treatment strategies for OA patients.

## Figures and Tables

**Table 1:** RT-qPCR primer sequences.

Gene	Sequences (5′–3′)	Product length (bp)
H-miR-182-5p	F: TTTG GCAATGGTAG AACTCACACCG	61
	R: GCTGTCAACGATACGCTACGTAA	
H-FGF9	F: GCTACAACGCTCCGCGA	118
	R:TCCATTGGCTTAGAACGGGT	
R-FGF9	F: ATACCTCGCCTAGTGTCTCCT	90
	R: TCACCTAAGGGAGCCATCAGA	
R-miR-182-5p	F: CGCGGGTCTAGCTGCC	60
	R: ACCGGTGTGAGTTCTACCAT	
H-U6	F: CTCGCTTCGGCAGCACA	94
	R: AACGCTTCACGAATTTGCGT	
H-*β*-actin	F: ACCCTGAAGTACCCCATCGAG	224
	R: AGCACAGCCTGGATAGCAAC	
R-5S	F: GCCTACAGCCATACCACCCGGAA	116
	R: CCTACAGCACCCGGTATCCCA	
R-*β*-actin	F: ACATCCGTAAAGACCTCTATGCC	223
	R: TACTCCTGCTTGCTGATCCAC	

H: human, R: rat.

## Data Availability

All data included in this study are available upon request by contact with the first author or corresponding author.
