# Correction to “Inhibition of miR‐182‐5p Targets FGF9 to Alleviate Osteoarthritis”

**DOI:** 10.1155/ancp/9879754

**Published:** 2026-08-24

Y. Sun, S. Su, M. Li, and A. Deng, “Inhibition of miR‐182‐5p Targets FGF9 to Alleviate Osteoarthritis,” *Analytical Cellular Pathology*, 2023, 5911546, https://doi.org/10.1155/2023/5911546.

In the article, concerns have been reported on PubPeer [1] regarding the apoptotic measurements. The authors maintain that the results and conclusions are correct; however, they wish to update Figures 2 and 5 and the methods section to provide further clarification. Specifically, in Figures 2d and 5g, the *Y*‐axis of the bar charts should be corrected from “Apoptosis (%)” to “Total Apoptotic Cells (Annexin V+) (%).”

Figure 2miR‐182‐5p inhibited chondrocyte proliferation and promoted apoptosis. (a) The gene expression of miR‐182‐5p. (b) CCK8 was used to determine the cell viability. (c) Photos and analysis of plate cloning of each group of cells. Flow cytometer was utilized to test the (d) apoptosis and (e) cell cycle. (f) The expression of inflammatory factors (TNF‐α, IL‐6, and IL‐8) was evaluated by ELISA.  ^∗^ *p* < 0.05 vs. control. ^#^ *p* < 0.05 vs. NC.

**Figure:**
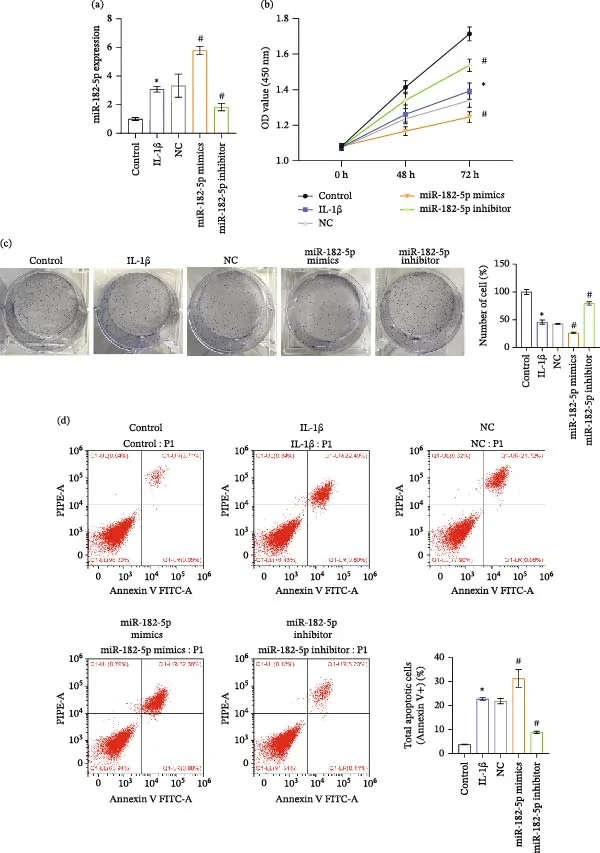

**Figure:**
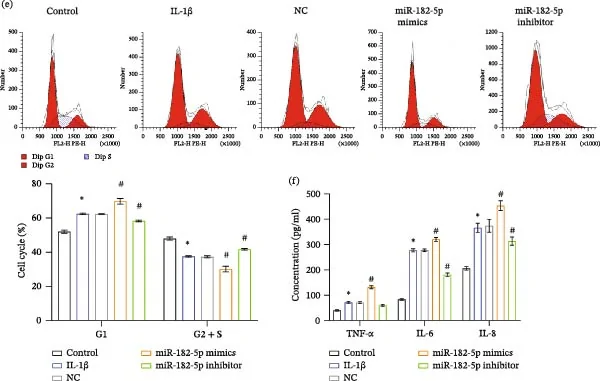

Figure 5miR‐182‐5p targeting FGF9 inhibited chondrocyte proliferation and promoted apoptosis. The gene expression of (a) miR‐182‐5p and (b) FGF9. (c) The protein expression of FGF9. (d) Cell viability was monitored by CCK8. (e) Plate cloning of cells. The (f) cell cycle and (g) apoptosis were captured by flow cytometry. (h) The expression of inflammatory factors (TNF‐α, IL‐6, and IL‐8) was evaluated by ELISA. ^#^ *p* < 0.05 vs. mimics NC. ^&^ *p* < 0.05 vs. miR‐182‐5p mimics + oe‐NC.

**Figure:**
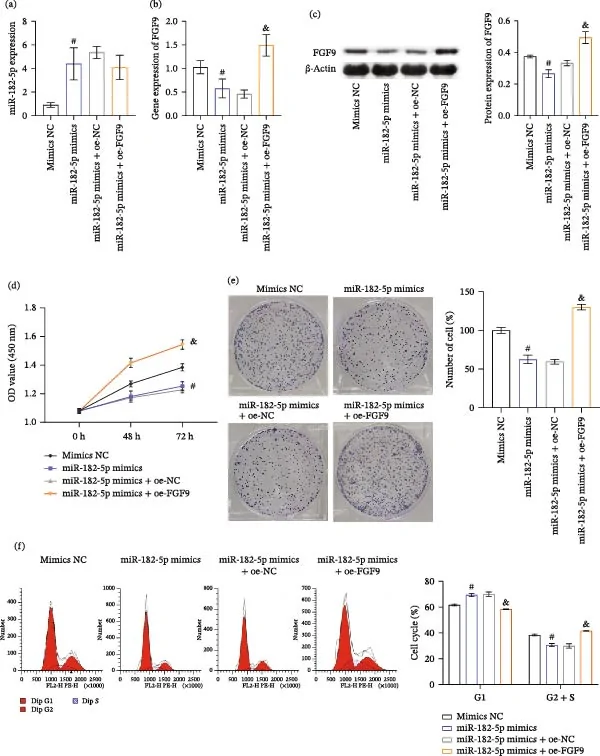

**Figure:**
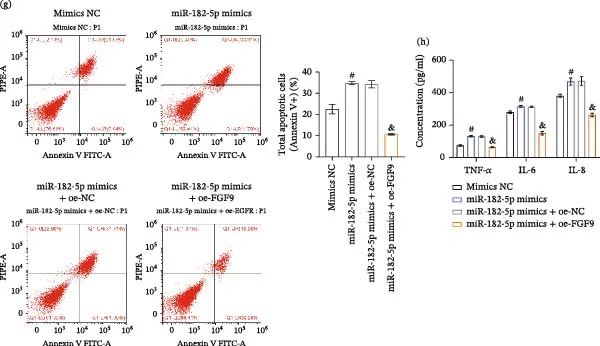

The correct Figures 2 and 5 are shown below:

Lastly, the following sentence should be added to the Materials and Methods section:

“For quantification, the total apoptotic cell percentage was defined as the sum of early apoptotic (Annexin V+ PI−) and late apoptotic/secondary necrotic (Annexin V+ PI+) cell populations.”

We apologize for these errors.

## References

[1] tanganyikensis I. , Inhibition of miR-182-5p Targets FGF9 to Alleviate Osteoarthritis, *PubPeer*, 2024, https://pubpeer.com/publications/F9E4DEAE257C5A5EB5DEE84218C3F9. 10.1155/2023/5911546 PMC10076120 37035017

